# Wettability Assessment of Hydrophobized Granular Solids: A Rheological Approach Using Surfactant Adsorption

**DOI:** 10.3390/ma18061305

**Published:** 2025-03-16

**Authors:** Xilena Villegas Arcos, Juliet Daniela Blanco Mayorga, Arlex Chaves-Guerrero, Ronald Mercado

**Affiliations:** 1 Grupo de Investigación en Fenómenos Interfaciales, Reología y Simulación de Transporte—FIRST, Universidad Industrial de Santander, Bucaramanga 680002, Colombiaachaves@uis.edu.co (A.C.-G.); 2 Grupo de Investigación en Recobro Mejorado—GRM, Universidad Industrial de Santander, Bucaramanga 680002, Colombia

**Keywords:** wettability, hydrophobization, granular solids, suspensions, surfactant, rheology

## Abstract

The wettability of granular solids is a critical parameter in numerous industrial applications, including enhanced oil recovery, advanced material coatings, and nanotechnology. However, traditional methods for assessing wettability, such as contact angle measurements, face significant challenges when applied to heterogeneous or porous solids. This study proposes a rheological methodology as an alternative approach to determine the wettability of granular solids, focusing on bentonite clay modified via sodium dodecylbenzene sulfonate adsorption. Aqueous and oily suspensions of bentonite with varying degrees of hydrophobicity were characterized using viscosity measurements, oscillatory amplitude sweeps, and thixotropic recovery tests. For the system under study, a bentonite concentration of 8% ensures optimal rheological behavior. Furthermore, the adsorption isotherm provides a reliable means of determining varying degrees of solid coverage. The results demonstrated clear correlations between surface coverage and rheological behavior, with increasing hydrophobicity leading to reduced viscosity and viscoelasticity in aqueous systems and a shift toward Newtonian flow behavior in oily systems. These findings were supported by traditional contact angle measurements, which confirmed the relationship between surfactant adsorption and enhanced hydrophobicity. The proposed rheological methodology overcomes the limitations of conventional wettability assessments and provides a new approach for characterizing and optimizing the interfacial properties of particulate systems. This work has broad implications across industries such as petroleum, coatings, and material science, offering a novel pathway for designing systems with tailored wettability and flow characteristics.

## 1. Introduction

The degree of hydrophobicity of a solid is a critically important property due to its broad range of applications across various fields. Modifying the wettability of solid surfaces has profound implications in industries such as petroleum, where enhanced oil recovery (EOR) is a key objective, as well as in the textile sector, infrastructure protection (such as coatings for solar panels and satellites), and the materials industry [1,2]. Within the materials field, superhydrophobic surfaces have gained significant attention for their potential applications in self-cleaning coatings, anti-corrosion layers, and advanced nanotechnologies [3].

The study of wettability and its modulation remains a dynamic area of research given its importance in both fundamental science and technological innovation. Recent advancements in surface science have provided valuable insights into the molecular-level interactions governing wettability, particularly in systems involving surfactants and mixed-phase media. Nonetheless, the intricate interplay of surface roughness, chemical heterogeneity, and interfacial energy continues to present substantial challenges to achieving accurate wettability characterization [4]. These challenges are particularly pronounced in granular and porous solids, where traditional methods often fail to adequately capture the complex behavior of such systems under realistic conditions.

Despite numerous studies on the phenomena influencing wettability, establishing clear and universal relationships between wettability and variables such as temperature, solid type, chemical composition, and the presence of surfactants remains a significant challenge. For instance, in EOR processes, the alteration of rock wettability is a pivotal factor [5]. Surfactants are frequently employed to modify the interactions between oil, water, and mineral surfaces, thereby exerting a substantial influence on the efficiency of oil recovery [6].

A direct measure of wettability is the contact angle, which quantifies the interaction between a liquid and a solid surface, providing valuable information about surface energy, roughness, and heterogeneity. In an idealized system, the contact angle is defined as the angle formed at the interface between two immiscible phases on a smooth, flat, and chemically homogeneous solid surface [7]. However, this ideal definition becomes problematic when applied to granular solids, where heterogeneity and porosity can introduce significant discrepancies between the measured contact angle and the actual degree of hydrophobicity. This challenge is particularly pronounced in porous materials such as clays, limestone, and silica, where variations in surface chemistry and roughness complicate precise measurements [8]. Furthermore, the complexity of the contact angle and the underlying physicochemical phenomena remain incompletely understood [9].

The wettability of a solid surface is directly related to its hydrophobization degree, which describes the extent to which the surface repels water. This relationship is typically quantified using the contact angle (θ) of a water droplet on the solid surface. Hydrophilic surfaces (low hydrophobization degree) have a high affinity for water, resulting in low contact angles (θ < 90°) [10]. Conversely, hydrophobic surfaces (high hydrophobization degree) repel water, leading to high contact angles (θ > 90°) [11]. Hydrophobization can be achieved by adsorbing surfactants, modifying functional groups, or grafting hydrophobic molecules onto the surface, reducing its attraction to water molecules [12].

Several methods have been described in the literature for evaluating the contact angle, including the capillary rise method and the sessile drop technique, among others [13,14]. However, when applied to heterogeneous materials such as granular solids, these methods often encounter additional challenges. Surface roughness, porosity, and chemical heterogeneity can introduce errors and lead to substantial variability in the measurements [15]. Numerous studies have highlighted potential sources of error in these techniques as well as the influence of experimental conditions and material properties on the reliability of the results obtained.

The determination of wettability in particulate solids and non-ideal surfaces, such as those found in oil reservoirs, is particularly critical due to the inherent roughness and heterogeneity of minerals like clay, silica, and limestone. These factors make it challenging to obtain precise contact angle measurements, thereby complicating the reliable estimation of a solid’s hydrophobicity. In oil reservoirs, the wettability of mineral surfaces plays a pivotal role in governing the distribution and flow of fluids, making it a key parameter for enhancing oil recovery and understanding surface chemistry interactions in subsurface environments [16].

Understanding the rheological properties of suspensions formed with granular solids is essential for optimizing processes across the petroleum, coatings, and materials industries. The rheology of such systems is heavily influenced by the wettability of the suspended particles, which impacts particle–particle interactions, aggregation, and dispersion stability. For example, clay suspensions with varying degrees of hydrophobicity exhibit distinct flow behaviors, ranging from Newtonian to highly shear-thinning or viscoelastic responses [17,18]. Despite its significance, the quantitative relationship between hydrophobicity and rheological behavior remains insufficiently explored, limiting the ability to predict and control these systems for industrial applications. Only a few studies have examined the effect of surface chemistry on the rheological behavior of suspensions, typically focusing on yield stress at varying pH levels, such as the works of Scales et al. [19] and Leong [20]. However, the scope and objectives of these studies differ significantly from those of the present work.

Beyond traditional applications, this research has broader implications for the design of advanced materials with tailored surface properties. Hydrophobically modified clay particles, for instance, could serve as templates for developing functionalized coatings or as additives in nanocomposites to enhance mechanical strength and barrier properties. Therefore, the objective of this work is to propose a novel methodology for determining the degree of wettability in granular solids. The approach involves preparing clay particles suspended in a continuous phase (whether oily or aqueous) to form a suspension, enabling the characterization of wettability through rheological analysis.

Wettability can thus be evaluated by comparing the rheological response, specifically in terms of viscosity and viscoelasticity, to contact angles. This first approach not only enhances the understanding of hydrophobicity in granular solids but also offers practical applications across various industries, including enhanced oil recovery, surface coatings, and advanced materials engineering.

## 2. Materials and Methods

The methodology involved preparing suspensions of bentonite particles with varying degrees of hydrophobicity achieved by adsorbing sodium dodecylbenzene sulfonate (SDBS) at different concentrations. Adsorption isotherms were utilized to quantify surface coverage and analyze adsorption behavior, with comparisons made to established theoretical models. Rheological tests, including viscosity measurements and oscillatory amplitude sweeps at a fixed frequency, were conducted on aqueous and oleic suspensions to assess the influence of surface coverage on viscosity and viscoelastic properties. These results were compared with measurements obtained using the traditional contact angle method to validate the effectiveness of rheological characterization as an alternative approach for determining the wettability of particulate solids.

Materials. Paraffin oil (supplied by VWR International, Radnor, PA, USA) was used as the oily phase for suspension preparation. Its viscosity at 25 °C is 17 mPa·s; and its boiling point rank is reported as 300–500 °C by the supplier. Sodium dodecylbenzene sulfonate (SDBS) with 99.9% purity (obtained from Aldrich) served as the amphiphilic molecule. This compound contains a chromophore group composed of benzene rings with a conjugated double bond system, enabling absorption of electromagnetic radiation in the ultraviolet (UV) range with a maximum absorbance wavelength of 224 nm. This property was utilized to quantify SDBS concentration in solution via UV spectroscopy. The critical micelle concentration (CMC) of SDBS was determined to be 0.1%wt (0.001 g/mL) using the tensiometer method.

The solid used in this study was bentonite clay (provided by Acofarma S.A., Madrid, Spain), a montmorillonite-type clay with hydrophilic properties. Due to its structure, this clay exhibits a high capacity to adsorb anionic compounds. Its specific surface area, measured using the Brunauer–Emmett–Teller (BET) method, was 65.26 m^2^/g. The average particle diameter was determined to be approximately 1.06 μm, with a narrow monomodal size distribution. Prior to use, the clay was thoroughly washed with distilled water and dried.

Formulation of Suspensions. In this study, two types of system were prepared: aqueous and oily suspensions. The formulation process aimed to determine the optimal solid-to-liquid ratio through rheological testing. Suspensions of bentonite in distilled water and bentonite in paraffin oil were prepared at various weight/volume concentrations (10%, 8%, 6%, and 4%). The suspensions were stirred at 20 rpm for 45 min to ensure uniform particle distribution before undergoing rheological characterization.

Zeta Potential and Particle Size Measurements. Particles sizes were determined using dynamic light scattering (DLS) with a LiteSizer 700 instrument (Anton Paar, Graz, Austria). Measurements were performed at a scattering angle of 180°, maintaining a controlled temperature of (25.0 ± 0.1) °C. The Zeta potential was also determined using this equipment at the same conditions. 

**Figure 1:**
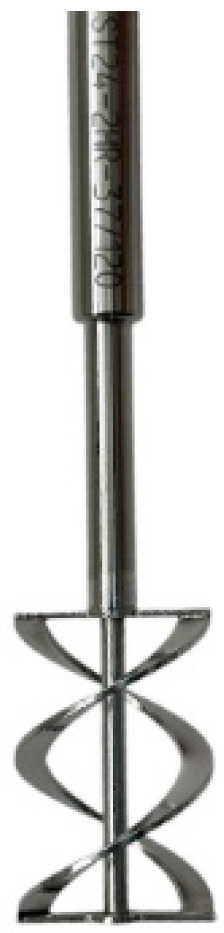
Helical ribbon geometry.

Rheological Characterizations. Rheological measurements were performed using an Anton Paar (Graz, Austria) MCR-302 rheometer equipped with RheoCompass software version 1.19. The tests utilized a Couette geometry comprising a static outer cup with a 40 mL volume and an inner double-helix spindle (ST24-2HR-37/120, Anton Paar, Figure 1). This geometry was selected to ensure effective dispersion of solid particles, as it is highly recommended for suspension measurements [21,22]. All tests were conducted at a constant temperature of 25 °C.

For viscosity curves, shear rate sweeps were performed from 100 s^−1^ to 0.1 s^−1^ using a logarithmic ramp. Additionally, oscillatory amplitude sweeps were conducted with strain amplitudes ranging from 0.01% to 100% at a constant angular frequency of 10 rad/s. To evaluate the thixotropic behavior of the suspensions, an oscillatory-rotational-oscillatory (ORO) test was carried out. The initial and final steps of the ORO test were conducted at a constant angular frequency of 10 rad/s and a strain amplitude of 0.1%, while the intermediate rotational step was performed at a shear rate of 1000 s^−1^. Each step in the ORO test was maintained for 300 s.

Quantification of Surfactant Concentration. The concentration of SDBS in aqueous solutions was determined via spectrometry using a Mapada P5 spectrophotometer (Shanghai, China) at the wavelength corresponding to the maximum absorbance of SDBS, i.e., 224 nm. 

Critical Micelle Concentration and SDBS Adsorption Isotherm on Bentonite. The critical micelle concentration of SDBS was assessed and determined by identifying the concentration at which further increases in SDBS no longer affected surface tension. Measurements were performed at 25 °C using a pendant drop tensiometer (Dataphysics, model OCA15, Filderstadt, Germany).

The adsorption of SDBS onto bentonite was quantified under controlled conditions at 25 °C. Samples were homogenized using a Thermo Scientific (Waltham, MA, USA) roller agitation system (model 8881003) at 20 rpm to ensure uniform mixing. Each sample consisted of bentonite and SDBS solution at a fixed mass-to-volume ratio of 1 g:10 mL. After an equilibration period of 24 h, solids were separated from the suspension using a centrifuge (Dathan Scientific, Wonju, South Korea, model Cef-50.6) operating at 3400 rpm for 20 min. The amount of adsorbed surfactant was calculated based on mass balance calculations. In all experiments, the temperature was maintained constant at 25 °C.

Contact Angle Measurement. Contact angle measurements were conducted using the sessile drop technique with a Dataphysics (Filderstadt, Germany) pendant drop tensiometer (model OCA15). Bentonite samples were treated with surfactant solutions of known concentrations to achieve varying degrees of hydrophobization, maintaining the same ratio used in the adsorption isotherm experiments (1 g bentonite per 10 mL solution). After 24 h of equilibration, the samples were centrifuged, and the resulting solid was compacted onto Petri dishes and dried completely to form a smooth and uniform surface. The compacted solids, treated with varying surfactant concentrations, exhibited different degrees of surface coverage based on the adsorption isotherm results.

Paraffin oil was used as the continuous phase, while water droplets were placed on the treated bentonite surfaces to measure contact angles. All measurements were conducted at 25 °C after allowing an equilibration period of 1 h.

All experimental tests were conducted three times, and the reported values represent the averages. Standard deviations are not included in the rheograms to prevent overlap with measurement markers. Instead, data points with appreciable error (i.e., standard deviation greater than 5%) are excluded from the rheograms.

## 3. Results and Discussion

### 3.1. Determination of the Optimal Solid Concentration in Suspensions 

**Figure 2:**
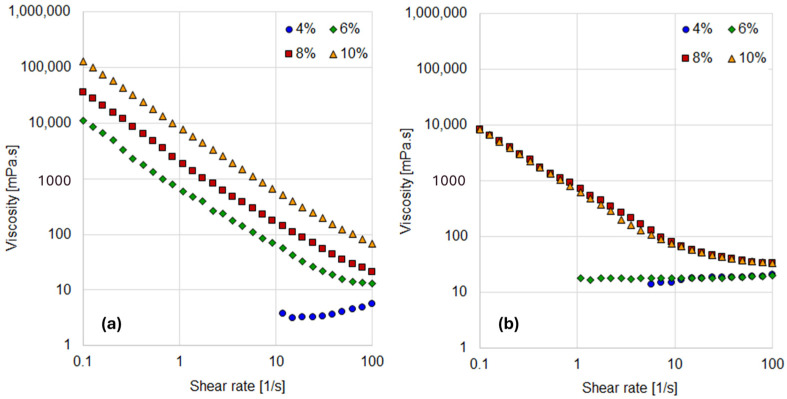
Viscosity curves as a function of solids content. (**a**) Aqueous suspensions. (**b**) Oily suspensions.

Four aqueous and oily dispersions with varying bentonite concentrations were analyzed, and the corresponding viscosity curves are presented in Figure 2.

For the aqueous suspensions, an increase in solid content resulted in a progressive rise in viscosity across all concentrations (Figure 2a). A shear-thinning behavior was observed for all suspensions except the 4% concentration. Shear thinning, a characteristic feature of highly concentrated bentonite suspensions, indicates that resistance to flow decreases as the deformation rate increases. This behavior is attributed to interparticle interactions such as double-layer repulsions [23].

At higher concentrations (8% and 10%), the viscosity profiles could be accurately described by the Power Law model, demonstrating a consistent non-Newtonian behavior. In contrast, the 6% suspension exhibited the onset of a constant viscosity plateau at high shear rates (above 50 s^−1^), which corresponds to the infinite-shear viscosity region.

Table 1 provides the rheological models and parameters for each suspension, highlighting the differences in flow behavior as a function of bentonite concentration.

As shown in Table 1, the consistency coefficient (K) increases with solids concentration, while the behavior index (n) remains relatively constant for suspensions exhibiting non-Newtonian behavior. In contrast, the 4% bentonite suspension does not exhibit significant deviations from Newtonian behavior, likely due to negligible interparticle interactions at this low concentration. Consequently, this concentration was excluded from further analysis.

These results are consistent with previous studies, which have demonstrated that a solid concentration has a significant impact on viscosity increase and the formation of Bingham plastic fluids [24,25].

Notable deviations from ideal Newtonian behavior are advantageous for subsequent tests, as they are more likely to reveal pronounced differences in rheological properties arising from varying degrees of particle hydrophobicity. Such distinctions are expected to be more prominent in systems where non-Newtonian behavior prevails, making higher concentrations of bentonite more suitable for evaluating the impact of hydrophobic modifications.

In oily suspensions, the viscosity curves for 4% and 6% exhibit purely Newtonian behavior (Figure 2b). In these cases, the Newtonian viscosity is higher than that observed in aqueous media, which can be attributed to the higher viscosity of the oil (17 mPa·s). At these lower bentonite concentrations, the interactions between particles and the oil medium are insufficient to induce significant non-Newtonian behavior. However, at 8% and 10% concentrations, shear-thinning behavior emerges, although the viscosity differences between these two suspensions are negligible. Table 1 provides the rheological models and parameters for the oily suspensions.

Although a similar behavior to that observed in aqueous suspensions was anticipated, the results suggest that the lower affinity between bentonite and oil introduces additional complexity to the rheological response. This complexity may be attributed to the aggregation of bentonite particles in the oily medium. Interestingly, the non-linear response observed at 6% and 8% concentrations in oily suspensions resembles the behavior seen in aqueous systems.

**Figure 3:**
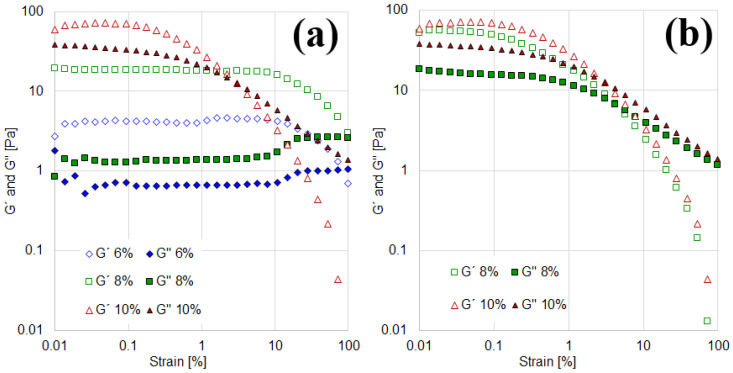
Amplitude sweeps as a function of solids content at 10 rad/s. (**a**) Aqueous suspensions. (**b**) Oily suspensions.

The particle interactions, influenced by their hydrophilicity, are central to this study. The rheological characterization aims to capture these interactions, as the viscosity curves of suspensions in both aqueous and oily media are highly dependent on them. To further confirm the findings from viscosity measurements, the viscoelastic behavior of the suspensions was analyzed through amplitude sweeps, with the results shown in Figure 3.

In aqueous suspensions, the amplitude sweeps (Figure 3a) demonstrate that at low strains, the elastic modulus (G′) is greater than the viscous modulus (G″), indicating a predominantly elastic structure capable of storing energy. As strain increases, the network structure collapses, leading to greater energy dissipation. Both moduli decrease continuously, but the drop in G′ is more significant than that in G″, resulting in a transition to viscous-liquid behavior at high strains.

For the 10% aqueous suspension, G′ and G″ exceed 10 Pa, with a crossover point (where G″ surpasses G′) observed at 2.2% strain. In contrast, the 8% and 6% suspensions exhibit lower moduli but maintain their solid-like structure over a wider strain range, with crossover points occurring at approximately 100% strain. This behavior is particularly notable as it indicates that the solid structure remains intact even under high shear strains.

For the 4% suspension, the results are not presented in Figure 3a, as no significant viscoelasticity was observed. This finding is consistent with the Newtonian behavior noted in the viscosity curves (Figure 2a).

On the other hand, oily suspensions also exhibit an elastic solid structure at low strains, as indicated by G′ being greater than G″ (Figure 3b). The two suspensions reported in this figure (8% and 10%) display similar viscoelasticity, with crossover points occurring at 4.2% and 3% strain, respectively. This behavior aligns with the viscosity curves observed in Figure 2b, confirming that both suspensions exhibit comparable shear-thinning and viscoelastic properties. Results for the 4% and 6% suspensions are not reported, as they exhibit negligible modulus values, consistent with the Newtonian behavior observed in the rotational tests. This can be attributed to their less-structured suspensions, characterized by more widely dispersed bentonite particles and weaker interactions (cohesive or electrostatic forces), which result in a lower network-forming capacity.

Based on the rotational and oscillatory results obtained thus far, the following partial conclusions can be drawn: For 4% suspensions, both aqueous and oily suspensions display Newtonian behavior and lack significant viscoelasticity. Consequently, they are excluded from further formulation processes. In the case of the 6% suspensions, they exhibit shear-thinning behavior and viscoelasticity only in aqueous suspensions. Lastly, in the case of the 8% and 10% suspensions, the two concentrations exhibit notable deviations from Newtonian behavior in both aqueous and oily media. While they show similar viscoelastic properties in oil, their aqueous counterparts demonstrate greater resistance to deformation despite having lower viscoelastic moduli.

**Figure 4:**
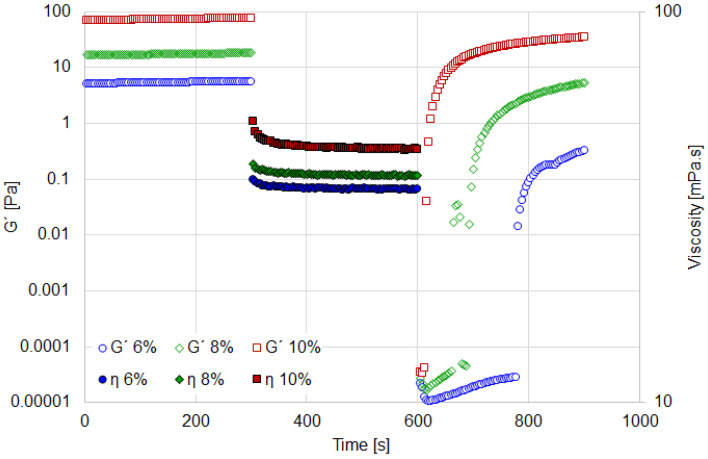
ORO test for aqueous suspension at several bentonite concentrations.

**Figure 5:**
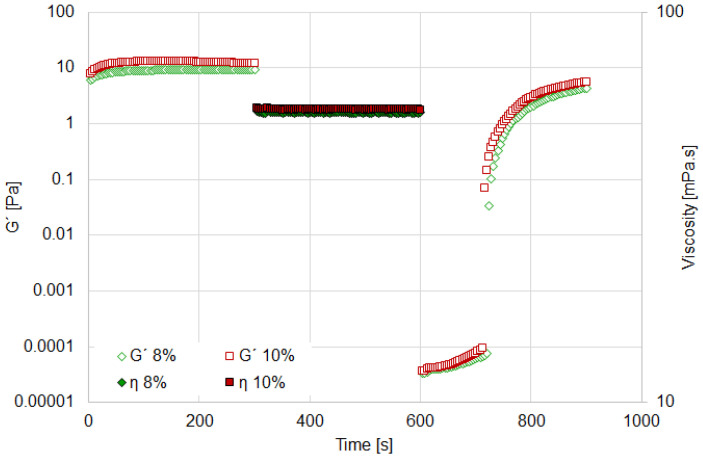
ORO test for oil suspension at several bentonite concentrations.

Thixotropy refers to the gradual decrease in viscosity under a constant shear force, with partial or complete recovery of the material’s initial structure when the shear is removed. The ORO test enables the characterization of recovery speed and rheological behavior. The results are presented in Figure 4 and Figure 5.

In aqueous media, all suspensions exhibit similar rheological behavior (Figure 4). A rapid recovery of the elastic modulus (G′) is observed after the application of rotational shear (set at a high speed of 1000 s^−1^ to ensure complete structural breakdown). Following a rest period of 300 s, the 10% suspension achieves a recovery of 45%, the 8% suspension recovers 29.4%, and the 6% suspension recovers 5.8% (Table 2).

On the other hand, as shown in Figure 5, the recovery behavior of the 8% and 10% suspensions in oily media is similar. After 300 s of rest, the recovery of the elastic modulus for these suspensions is 48% and 46.8%, respectively (Table 2). A rapid recovery of suspension structure is desirable, as it ensures precise and consistent rheological properties during both rotational and oscillatory characterizations. In other words, rapid recovery is critical for maintaining the intended rheological behavior, thereby ensuring the reliability of the suspensions under various testing conditions.

Based on these findings, the 8% suspension was selected to study the effect of particle hydrophobicity on rheological behavior. This concentration exhibits an optimal viscosity profile with significant deviations from Newtonian behavior in both aqueous and oily media. Furthermore, the 8% suspension demonstrates sufficiently rapid structural recovery along with notable viscoelasticity and resistance to shear strain.

### 3.2. Study of the Effect of Bentonite Hydrophobicity Degree on the Rheological Behavior of Suspensions

**Figure 6:**
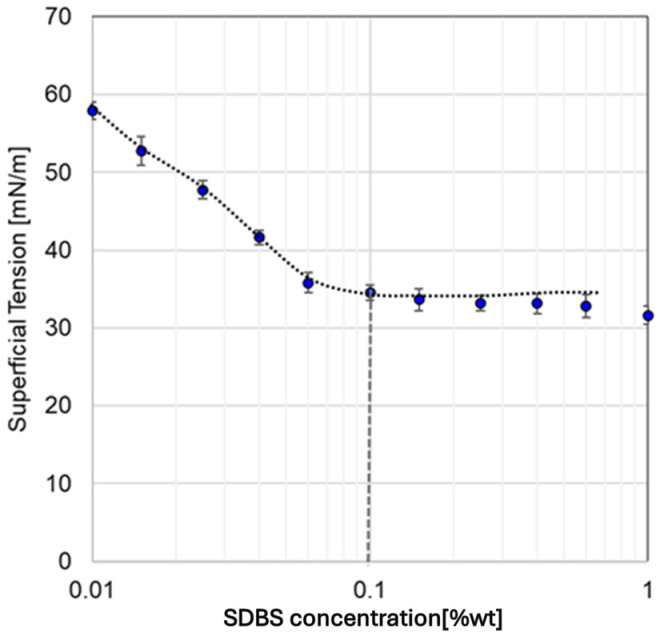
Superficial tension as a function of SDBS concentration.

The CMC of SDBS was determined from surface tension measurements, as shown in Figure 6. At 25 °C, the CMC was found to be 0.1%wt (2.87 mM), a value consistent with other reports obtained through conductivity methods [26,27]. At concentrations above the CMC, SDBS molecules form micelles, with their hydrophobic tails oriented inward and hydrophilic heads interacting with water, thereby stabilizing surface tension.

This behavior adheres to the Gibbs isotherm equation (Equation (1)), which correlates the reduction in surface tension with the adsorption of molecules at the interface. Initially, as the SDBS concentration increases, surface tension decreases due to the adsorption of surfactant molecules at the air–water interface. Once the CMC is surpassed, the surface tension remains constant because the interface becomes saturated with SDBS molecules, and excess surfactant molecules exist primarily in micellar form.

Using the Gibbs isotherm equation, the specific surface area of the surfactant molecules was determined to be 8.68 × 10^−10^ moles/cm^2^ (19.1 Å^2^/molecule). This parameter is particularly significant, as it enables the adsorption isotherm to be expressed in terms of surface coverage rather than the traditional ratio of surfactant moles adsorbed per unit mass of solid.

(1) $$T_{i}=\frac{1}{RT}\left(-\frac{d\gamma }{dLnC}\right)$$

In the equation above, Ti represents the excess interfacial concentration (moles/cm^2^), R denotes the universal gas constant, and T refers to the absolute temperature (K).

#### 3.2.1. Adsorption Isotherm Calculation

The adsorption isotherm was determined by quantifying the amount of SDBS adsorbed onto the solid as a function of its initial concentration, maintained at a constant temperature of 25 °C. This analysis provides insight into the varying levels of surface coverage achieved on the solid as a function of the equilibrium surfactant concentration/CMC ratio.

**Figure 7:**
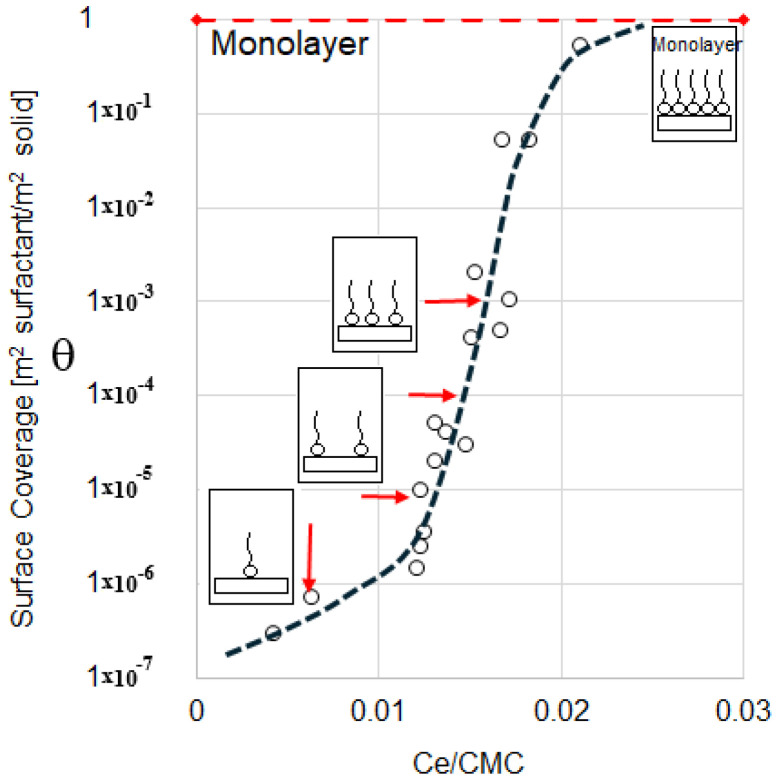
Adsorption isotherm of SDBS on bentonite at 25 °C. The surface coverage is presented as a function of equilibrium concentration.

In Figure 7, the isotherm is displayed on a normal-log scale, facilitating the observation of the full range of surface coverage levels. Upon evaluation, the behavior closely aligns with an S-type isotherm [28]. The dotted line represents the general trend of adsorption. Previous studies suggest that surfactant adsorption on solid surfaces in simple systems or those involving a single surfactant species can be described using a four-region or two-step model [29]. However, in this study, the x-axis range spans only one order of magnitude, as the aim is to control the hydrophobization of the bentonite surface while avoiding the formation of hemi-micelle structures. A quasi-vertical monomolecular adsorption step is observed, indicating that surface coverage increases with the concentration of surfactant (see Figure 7). Beyond the highest concentration considered in this study (0.021), a plateau is expected, followed by a second adsorption increase characterized by the formation of hemi-micelles when surface coverage exceeds one (θ > 1). This behavior is typical of the two-step adsorption model. Cases et al. describe similar phenomena in systems with strong adsorbate–adsorbent interactions [30].

Within the surfactant concentration range used in this study, only a monolayer of surfactant is formed. This outcome is particularly advantageous since bentonite particles could revert to hydrophilic behavior if a partial bilayer were to form on their surface. Based on these findings, the data in this study conform to a single-step adsorption isotherm. In the initial region, between 0.004 and 0.012 on the x-axis, only a limited number of molecules adsorb onto the bentonite surface. As the surfactant concentration increases, adsorption intensifies, with surface coverage rising by several orders of magnitude between 0.012 and 0.018. This trend continues until a concentration of 0.021 is reached, where the solid’s surface becomes half-saturated (θ = 0.51), which is marked by a noticeable change in the slope.

Beyond this concentration, no additional data were collected, for two reasons: first, the study does not focus on achieving higher surface coverage, and second, bentonite’s high adsorption capacity, attributable to its extensive surface area (65.3 m^2^/g), makes this unnecessary. In fact, a surfactant concentration approaching the solution’s saturation point was used to obtain the final data point on the curve. These results are consistent with previous studies [31,32]. However, in this work, the equilibrium concentration range is significantly broader than that reported in traditional studies. This broader range is particularly important, as it enables the assessment of coverage levels across several orders of magnitude, allowing for a more thorough investigation of their influence on the rheological behavior of suspensions of these hydrophobic solids.

In summary, the surfactant concentrations employed in this study do not extend to higher surface coverages. However, it is anticipated that beyond the CMC, further adsorption onto the bentonite surface could occur due to the formation of a second surfactant layer (hemi-micelles) driven by van der Waals interactions between the aliphatic tails of the surfactant molecules.

The surface coverage levels of the solid were defined based on the initial and equilibrium surfactant concentrations, as indicated by the red arrows on the adsorption isotherm (Figure 7). These concentrations, along with the corresponding surface coverage values, are summarized in Table 3. It is worth noting that higher coverage levels could not be achieved, a constraint arising from the high adsorption capacity of bentonite, as previously discussed.

#### 3.2.2. Influence of Surface Coverage on Rheological Behavior

**Figure 8:**
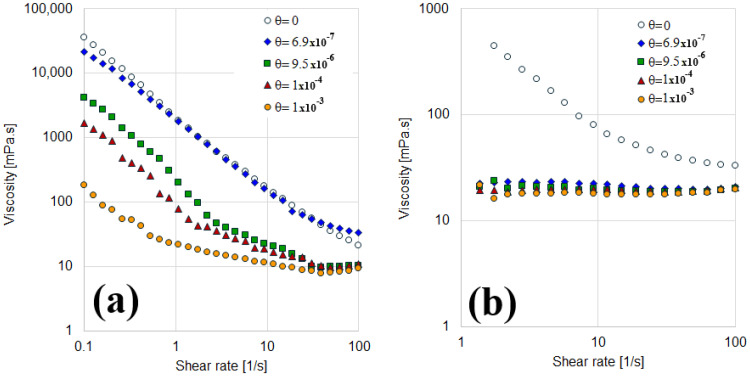
Viscosity curves as a function of particles coverage. (**a**) Aqueous suspensions. (**b**) Oily suspensions.

For aqueous suspensions, a clear trend is evident in Figure 8a: higher surface coverage leads to lower viscosity. Suspensions composed of fully hydrophilic solids (bentonite without adsorbed SDBS molecules) exhibit the highest viscosity, closely followed by the behavior of particles labeled as B (θ = 6.9 × 10^−7^). This high viscosity is attributed to the negative surface charge of bentonite particles, which induces strong electrostatic repulsions between particles, increasing the system’s viscosity. As SDBS adsorption increases, these repulsions are mitigated (see Table 3), and surface coverage rises, enhancing interparticle lubrication. This phenomenon occurs because the hydrophobic tails of the adsorbed surfactant molecules partially cover the particle surfaces, reducing direct interactions between them [33]. Consequently, electrostatic repulsions weaken, and hydrophobic interactions between the surfactant tails become dominant, resulting in a viscosity decrease. This behavior aligns with findings reported by Luckham and Rossi [23], which highlight how surfactant adsorption influences the electrical double layer and van der Waals interactions.

Interestingly, an associated reduction in ionic strength extends the range of electrostatic repulsions between particles. This can significantly increase viscosity at low shear rates, potentially leading to the formation of an apparent yield stress or infinite zero-shear viscosity [33].

The rheological behavior of the suspensions was modeled using the Sisko and Power Law equations, with the resulting parameters presented in Table 4. For hydrophobized particles, a viscosity plateau is observed at high shear rates, conforming to the Sisko model. In contrast, suspensions of uncoated particles (θ = 0) follow the Power Law model. These models are primarily specified to demonstrate two key aspects: first, that viscosity behavior is significantly influenced by the degree of solid coating, and second, that they could potentially serve as tools for establishing a pseudo-calibration curve. However, within the scope of this study, they have not been employed for this purpose. Instead, their use highlights the changes in rheological behavior induced by variations in surface coverage.

Importantly, the surface coverage evaluated in this study remains below unity (θ < 1), aligning with expectations. However, if coverage were to increase beyond this range, forming hemimicellar structures, the solid particles would become hydrophilic again. This would also increase the apparent particle size and solids concentration, leading to higher viscosity and significant deviations from Newtonian behavior. Such outcomes could compromise the effectiveness of the technique, emphasizing the need for a well-defined calibration system where surface coverage remains within the range (0 ≤ θ ≤ 1).

For oily suspensions, a distinct behavior is observed. The viscosity curves shift from shear-thinning to Newtonian across all surface coverage levels (Figure 8b). Fully hydrophilic particles (θ = 0) exhibit a larger pseudo-volume due to their water-wet nature, which prevents them from properly interacting with the oil phase. This behavior makes the system behave as a more concentrated suspension. However, minimal surfactant adsorption induces a wettability change, even at low surface coverage levels, reducing particle interactions and producing a consistent Newtonian response in suspensions containing non-purely hydrophilic solids.

If surface coverage were to exceed unity (θ > 1), further adsorption would likely increase viscosity due to the enhanced electrostatic repulsions resulting from additional surfactant coatings. In such cases, oily suspensions might serve as a more appropriate calibration system than aqueous ones.

As a preliminary conclusion at this stage, it can be stated that the rheological behavior of suspensions is significantly influenced by the continuous phase and the surface coverage of bentonite particles. In aqueous suspensions, an increase in SDBS adsorption reduces viscosity by diminishing electrostatic repulsion and promoting interparticle lubrication through hydrophobic interactions. Conversely, in oily suspensions, a transition from shear-thinning to Newtonian behavior is observed as fully hydrophilic particles occupy a greater pseudo-volume due to their incompatibility with the oil phase. Minimal surfactant adsorption modifies wettability, reducing particle interactions and leading to consistent Newtonian flow, highlighting the critical role of wettability in suspension rheology.

**Figure 9:**
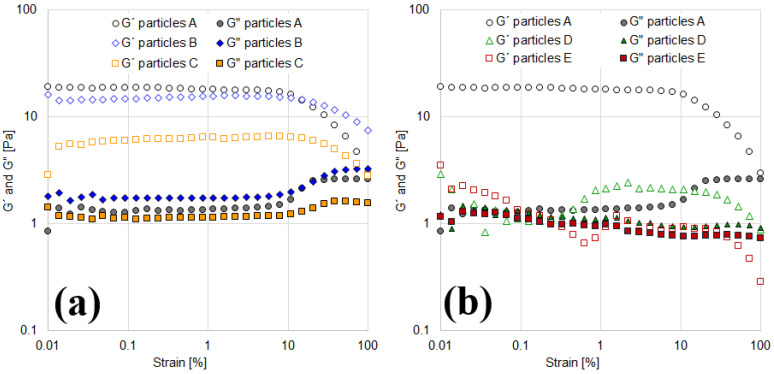
Oscillatory amplitude strain sweeps as a function of particles coverage. (**a**) Particles A, B, and C. (**b**) Particles A, D, and E.

Oscillatory amplitude sweep tests at a constant frequency (10 rad/s) were performed on both aqueous and oily suspensions (Figure 9). In aqueous suspensions, fully hydrophilic particles exhibited the highest viscoelasticity, with an elastic modulus (G′) exceeding 10 Pa, as shown in Figure 9a. This structured system is dominated by electrostatic repulsions. As surface coverage increases, the elastic modulus (G′) progressively decreases. For particles B (θ = 9.47 × 10^−6^) and C (θ = 1.01 × 10^−4^), the elastic (G′) and viscous (G″) moduli become comparable, with magnitudes around 1 Pa.

In these systems, the viscoelasticity of the formed structures is reduced, and the elastic component no longer dominates over the viscous one. Notably, no crossover points between G′ and G″ were observed within the studied shear strain range. This point, known as the yield stress, could serve as a calibration parameter for particles with varying surface coverage.

Interestingly, in suspensions with more hydrophilic particles (A, B, and C), the viscous modulus (G″) increases in magnitude from 10% strain onward (Figure 9a), indicating the formation of microcracks within the suspension structure. In contrast, suspensions with higher surface coverage (D and E) exhibit a decrease in G″ from 10% strain onward (Figure 9b).

In oily suspensions, viscoelasticity was negligible for all partially covered solids, as reflected in the viscosity curves (Figure 8b). Only fully hydrophilic particles exhibited measurable viscoelasticity, while both G′ and G″ remained negligible for the other systems.

Up to this point, it can be established that the rheological and viscoelastic behavior of suspensions is significantly influenced by the continuous phase and the surface coverage of bentonite particles. In aqueous suspensions, increasing SDBS adsorption reduces viscosity by attenuating electrostatic repulsions and enhancing interparticle lubrication through hydrophobic interactions. In contrast, oily suspensions transition from shear-thinning to Newtonian behavior, as surfactant adsorption alters wettability and reduces particle interactions.

Oscillatory tests confirm these trends, with hydrophobicity playing a critical role in modulating viscoelasticity. As surface coverage increases, the elastic component decreases, reflecting the reduced influence of electrostatic repulsions.

These findings validate the hypothesis that particulate solid wettability can be determined through rheological tests (rheometry). To further support these conclusions, a comparison with a traditional method, contact angle measurements, was conducted.

### 3.3. Contact Angle Comparisons

**Figure 10:**
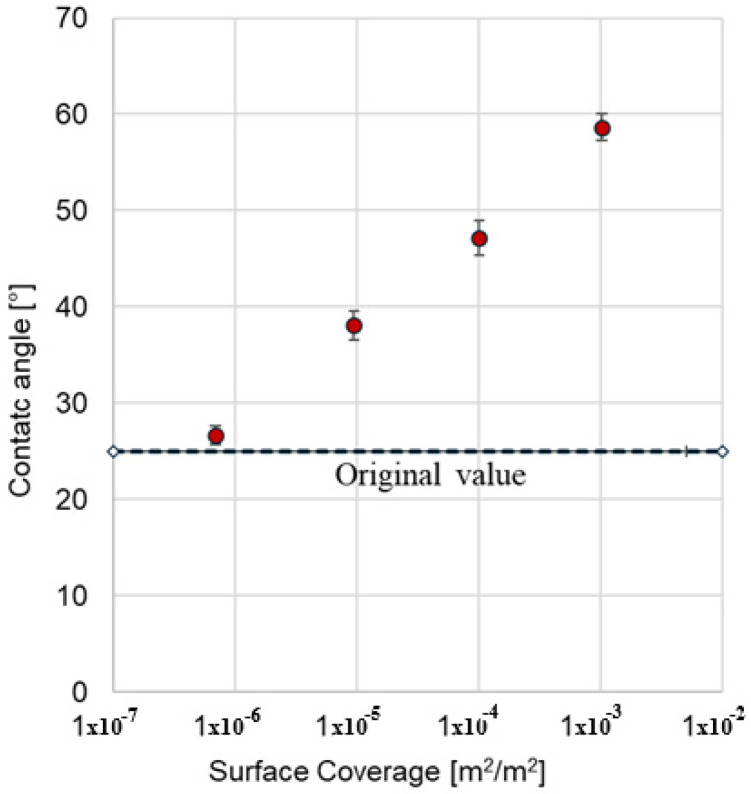
Contact angle as a function of a solid’s surface coverage.

The wettability of a solid by a liquid can be determined through contact angle measurements [34]. However, this technique is robust and reliable primarily when the solid is consolidated and completely smooth, yielding better results on flat, non-rough surfaces such as metal or glass plates. In this study, the contact angle was measured using a brine pendant drop on the surface of bentonite with varying surface coverages. The results, shown in Figure 10, reveal a direct correlation: higher SDBS coverage results in higher contact angles.

Interestingly, the figure suggests a linear relationship between the contact angle and the logarithm of the degree of coating. Beyond this observation, it is evident that wettability to water decreases as surfactant adsorption on the surface increases. As the coating increases, the surface becomes more hydrophobic, leading to higher contact angles.

A comparison between the rheological results and contact angle measurements confirms that increasing the surfactant coating enhances the hydrophobicity of bentonite, making the surface progressively less water-wet. This effect is reflected in reduced viscosity curves and elasticity alongside an increase in the measured contact angle.

### 3.4. Final Approach and Likely Limitations

Based on the methodology and results, a standard method for determining the hydrophobicity degree of bentonite can be established by combining rheological characterization with traditional contact angle measurements for validation.

The proposed approach begins with identifying the most reliable solid concentration, which is critical since most suspensions exhibit non-Newtonian behavior and rapid structural recovery. As an additional step, a calibration curve in terms of surface coverage should be prepared by varying the degree of hydrophobicity through the adsorption of a specific molecule (e.g., a surfactant such as sodium dodecylbenzene sulfonate) at controlled concentrations. The choice of molecule depends on the intended application. For instance, in the context of oil recovery involving unconsolidated particulate rocks, the solid could be treated with asphaltenes, resins, fatty acids, or other compounds to achieve the desired degree of hydrophobicity. In these cases, the preparation of oily suspension is probably more suitable.

The next step involves performing rheological tests, specifically, measuring viscosity across a range of shear rates to evaluate the flow behavior and detect changes in shear-thinning properties as hydrophobicity increases. Complementary oscillatory amplitude sweep tests at a fixed frequency are also desirable to assess viscoelastic properties, particularly the elastic (G′) and viscous (G″) moduli, as indicators of interparticle interactions.

To further validate the rheological method, additional techniques may be incorporated. In this study, the contact angle method was selected because hydrophobized clay forms a smooth and uniform surface suitable for measurement. This feature is unique to clays due to their small particle size and hydration properties. This characteristic allows for a more reliable assessment of wettability. However, for other materials, such as silicates, it may not be possible to construct surfaces with the required smoothness for reliable measurement. In these cases, adsorption isotherms remain the primary tool for quantifying surface coverage and attempting to correlate this parameter with solid wettability.

The final step involves establishing a correlation and validation framework by comparing rheological results (such as reductions in elastic modulus and viscosity) with confirming values such as contact angle measurements, if possible. This comparison helps establish a quantitative relationship between these parameters and the solid coverage degree. This integrated methodology combines the sensitivity of rheology to detect subtle changes in particle interactions with a widely recognized method for assessing surface hydrophobicity. The dual approach ensures robustness and reliability in determining the hydrophobicity degree of particulate solids, but the limitations associated with contact angle measurements for granular materials due to their inherent surface roughness are acknowledged.

Alternative systems are currently being studied, as the system selected for this initial work may not fully represent more realistic scenarios involving larger particles and other relevant complexities. Additionally, a significant constraint observed in this work is the maximum coverage degree attained. If a second surfactant layer forms, the particles may regain hydrophilicity, potentially leading to an increase in viscosity and elasticity. This observation underscores the need to establish a more reliable approach for constructing a “calibration curve”, tailored to specific solid–solvent interactions.

Additionally, when working with different systems, the initial stage will likely allow for the estimation of the higher suspension concentrations necessary to observe deviations from Newtonian behavior. This is primarily due to two factors, larger particle size and a reduced electrokinetic potential, both of which can diminish interparticle interactions. Additionally, the recovery time may impose a limitation on the methodology. If the system exhibits pronounced thixotropic behavior and the structural recovery kinetics are slow, reproducibility issues may arise in both viscosity curves and oscillatory tests.

Another critical factor can be associated with solid heterogeneity, which can lead to complex adsorption mechanisms, making the determination of surface coverage from adsorption isotherms less straightforward. Likewise, the nature of the surfactant plays a crucial role in governing the adsorption mechanism, and for greater consistency, monomolecular surfactant systems can consequently be recommended.

This study can be regarded as an initial step toward establishing a standardized methodology for assessing the degree of hydrophobization or wettability of particulate solids.

## 4. Conclusions

The rheological behavior of the suspensions as a function of solids concentration aligns with expectations, as does the adsorption isotherm of SDBS on bentonite. However, to the best of our knowledge, no prior studies have specifically investigated the influence of the degree of hydrophobization on viscosity curves or oscillatory tests. Only a limited number of studies have explored the effect of surface chemistry on the rheological behavior of suspensions, typically focusing on yield stress variations with pH.

The findings of this research demonstrate that the degree of hydrophobicity, modulated by surfactant adsorption, significantly influences the rheological properties of bentonite suspensions. In aqueous suspensions, increasing surface coverage results in a progressive reduction in viscosity and viscoelastic modulus, attributed to the attenuation of electrostatic repulsions and enhanced interparticle lubrication.

Hydrophobic modifications in aqueous suspensions induce a notable transition from shear-thinning to near-Newtonian behavior accompanied by a decrease in structural rigidity, as reflected in the lower elastic (G′) and viscous (G″) moduli. In contrast, oily suspensions exhibit distinct behavior, with Newtonian flow characteristics emerging at all hydrophobicity levels beyond the fully hydrophilic state. This divergence highlights the influence of the continuous phase’s polarity on particle–particle interactions.

Thixotropic recovery tests confirm that higher solid concentrations (e.g., 8%wt) efficiently regain their structure, making them suitable for studying hydrophobic modifications. Rapid recovery ensures reliable rheological behavior during repeated deformation cycles.

The traditional contact angle method corroborated the rheological findings, revealing a proportional increase in contact angle with the logarithm of the degree of surfactant coverage. This dual approach underscores the robustness of the proposed methodology for assessing wettability. Rheology emerges as a sensitive, quantitative tool for evaluating particulate solids’ wettability, overcoming the limitations of conventional methods on heterogeneous and porous materials.

This study represents an initial step toward understanding the relationship between surface coverage and wettability in granular solids using rheological methods. However, the system examined here may not fully reflect more realistic conditions, such as those involving larger particles or more complex solid–fluid interactions. Additionally, a key limitation observed is the maximum coverage degree attained, beyond which the formation of a secondary surfactant layer may induce a reversal in hydrophobicity, potentially increasing viscosity and elasticity. These findings highlight the need for a more robust approach, particularly the development of a reliable “calibration curve” tailored to specific solid–solvent systems. Ongoing research aims to address these challenges by exploring alternative systems and refining the methodology to improve its applicability to a broader range of materials.

This research lays the foundation for broader applications, suggesting that similar methodologies can be extended to more complex systems such as composite materials, oilfield rocks, or modified particles with multiple surfactants. Future investigations will focus on exploring the interplay between surface chemistry, interfacial forces, and rheological properties to enhance predictive capabilities.

## Figures and Tables

**Table 1:** Rheological models’ parameters as a function of the concentration of solids obtained from viscosity curves.

Suspension Type	Solids Content, %wt	Model	Equation	Consistency Coefficient k, mPa·s^n^	Behavior Index, n	R^2^
Aqueous	4	Newton	$\sigma =3.98\dot{\gamma }$	-	1	-
Aqueous	6	Sisko	$\eta =\eta_{\infty }+k{\left|\dot{\gamma }\right|}^{n-1}$	697.8	−0.167	0.9995
Aqueous	8	Power Law	$\eta =k{\left|\dot{\gamma }\right|}^{n-1}$	2330.2	−0.100	0.9980
Aqueous	10	Power Law	$\eta =k{\left|\dot{\gamma }\right|}^{n-1}$	8828.2	−0.115	0.9980
Oily	4	Newton	$\sigma =17.93\dot{\gamma }$	-	1	-
Oily	6	Newton	$\sigma =18.22\dot{\gamma }$	-	1	-
Oily	8	Sisko	$\eta =\eta_{\infty }+k{\left|\dot{\gamma }\right|}^{n-1}$	660.39	−0.1567	0.9952
Oily	10	Sisko	$\eta =\eta_{\infty }+k{\left|\dot{\gamma }\right|}^{n-1}$	587.35	−0.1963	0.9991

**Table 2:** Suspensions recovery according to an ORO test.

Solids Content, %wt	Aqueous Recovery, %	Oily Recovery, %
10	45.0 ± 0.8	46.8 ± 0.9
8	29.4 ± 0.5	48.0 ± 0.7
6	5.8 ± 0.3	-

**Table 3:** Solid surface coverage as a function of the initial surfactant concentration.

Particles	Initial Surfactant Concentration, %wt	Equilibrium Surfactant Concentration/CMC	Bentonite Surface Coverage, θ, m^2^/m^2^	pZ, mV
A	0	0	0	−41.4 ± 0.5
B	0.002	6.37 × 10^−3^	6.9 ± 0.3 × 10^−7^	−40.0 ± 0.5
C	0.020	1.23 × 10^−2^	9.5 ± 0.5 × 10^−6^	−39.1 ± 0.5
D	0.200	1.27 × 10^−2^	1.00 ± 0.05 × 10^−4^	−37.6 ± 0.4
E	2	1.73 × 10^−2^	1.00 ± 0.04 × 10^−3^	−36.4 ± 0.3

**Table 4:** Rheological model parameters as a function of surface coverage obtained from viscosity curves.

Suspension Type	Surface Coverage, cm^2^/cm^2^	Model	Equation	Consistency Coefficient k, mPa·s^n^	Behavior Index, n	R^2^
Aqueous	0	Power Law	$\eta =k{\left|\dot{\gamma }\right|}^{n-1}$	2330.2	−0.100	0.9980
Aqueous	6.9 × 10^−7^	Sisko	$\eta =\eta_{\infty }+k{\left|\dot{\gamma }\right|}^{n-1}$	1788.1	−0.159	0.9983
Aqueous	9.5 × 10^−6^	Sisko	$\eta =\eta_{\infty }+k{\left|\dot{\gamma }\right|}^{n-1}$	221.9	−0.310	0.9912
Aqueous	1 × 10^−4^	Sisko	$\eta =\eta_{\infty }+k{\left|\dot{\gamma }\right|}^{n-1}$	102.5	−0.153	0.9852
Aqueous	1 × 10^−3^	Sisko	$\eta =\eta_{\infty }+k{\left|\dot{\gamma }\right|}^{n-1}$	17.9	0.192	0.9817
Oily	0	Sisko	$\eta =\eta_{\infty }+k{\left|\dot{\gamma }\right|}^{n-1}$	660.39	−0.1567	0.9952
Oily	6.9 × 10^−7^	Newton	$\sigma =21.7\dot{\gamma }$	-	-	-
Oily	9.5 × 10^−6^	Newton	$\sigma =19.9\dot{\gamma }$	-	-	-
Oily	1 × 10^−4^	Newton	$\sigma =19.4\dot{\gamma }$	-	-	-
Oily	1 × 10^−3^	Newton	$\sigma =18.3\dot{\gamma }$	-	-	-

## Data Availability

The original contributions presented in this study are included in the article. Further inquiries can be directed to the corresponding author.

## Abbreviations

The following abbreviations are used in this manuscript:

EOR	Enhanced oil recovery
SDBS	Sodium dodecylbenzene sulfonate
CMC	Critical micelle concentration
ORO	Oscillatory-rotational-oscillatory
